# Genomic characterisation of the new *Dickeya fangzhongdai* species regrouping plant pathogens and environmental isolates

**DOI:** 10.1186/s12864-018-5332-3

**Published:** 2019-01-11

**Authors:** Špela Alič, Jacques Pédron, Tanja Dreo, Frédérique Van Gijsegem

**Affiliations:** 1 National Institute of Biology, Vecna pot 111, SI-1000 Ljubljana, Slovenia; 2 Jozef Stefan International Postgraduate School, Jamova 39, SI-1000 Ljubljana, Slovenia; 3 Institute of Ecology and Environmental Sciences of Paris, Sorbonne Universités, UPMC Univ Paris 06, Diderot Univ Paris 07, UPEC Univ Paris 12, CNRS, INRA, IRD, 4 Place Jussieu, 75005 Paris, France

**Keywords:** T5SS, T6SS, NRPS/PKS, Zeamine, oocydin A, Plant-bacteria interactions, Plasmid, *Dickeya fangzhongdai*

## Abstract

**Background:**

The *Dickeya* genus is part of the *Pectobacteriaceae* family that is included in the newly described *enterobacterales* order. It comprises a group of aggressive soft rot pathogens with wide geographic distribution and host range. Among them, the new *Dickeya fangzhongdai* species groups causative agents of maceration-associated diseases that impact a wide variety of crops and ornamentals. It affects mainly monocot plants, but *D. fangzhongdai* strains have also been isolated from pear trees and water sources. Here, we analysed which genetic novelty exists in this new species, what are the *D. fangzhongdai*-specific traits and what is the intra-specific diversity.

**Results:**

The genomes of eight *D. fangzhongdai* strains isolated from diverse environments were compared to 31 genomes of strains belonging to other *Dickeya* species. The *D. fangzhongdai* core genome regroups approximately 3500 common genes, including most genes that encode virulence factors and regulators characterised in the *D. dadantii* 3937 model strain. Only 38 genes are present in *D. fangzhongdai* and absent in all other *Dickeyas*. One of them encodes a pectate lyase of the PL10 family of polysaccharide lyases that is found only in a few bacteria from the plant environment, soil or human gut. Other *D. fangzhongdai*-specific genes with a known or predicted function are involved in regulation or metabolism.

The intra-species diversity analysis revealed that seven of the studied *D. fangzhongdai* strains were grouped into two distinct clades. Each clade possesses a pool of 100–150 genes that are shared by the clade members, but absent from the other *D. fangzhongdai* strains and several of these genes are clustered into genomic regions. At the strain level, diversity resides mainly in the arsenal of T5SS- and T6SS-related toxin-antitoxin systems and in secondary metabolite biogenesis pathways.

**Conclusion:**

This study identified the genome-specific traits of the new *D. fangzhongdai* species and highlighted the intra-species diversity of this species. This diversity encompasses secondary metabolites biosynthetic pathways and toxins or the repertoire of genes of extrachromosomal origin. We however didn’t find any relationship between gene content and phenotypic differences or sharing of environmental habitats.

**Electronic supplementary material:**

The online version of this article (10.1186/s12864-018-5332-3) contains supplementary material, which is available to authorized users.

## Background

Soft rot *Pectobacteriaceae* are *Enterobacterales* responsible for considerable economic losses in several important crops and ornamental plants [1–3]. Their virulence is mainly due to the production and secretion of a battery of plant cell wall degrading enzymes (PCWDEs) that cause maceration of the plant tissue; however, several other virulence factors have also been characterized [2, 4]. These bacteria often exhibit a very broad host range, and recent outbreaks in potato, for example, resulted from the action of a cohort of bacteria belonging to different *Pectobacteriaceae* species in a complex population dynamics history [5]. The *Pectobacteriaceae* family includes two genera comprising soft rot bacteria, *Pectobacterium* and *Dickeya*. The *Dickeya* genus was formed in 2005 by the reclassification of former *Erwinia chrysanthemi* into six species [6]. It has recently undergone multiple phylogenetic changes, including the addition of three new species, *Dickeya solani* [7], *Dickeya aquatica* [8] and, more recently, *Dickeya fangzhongdai* [9].

The description of this last new species was based on three isolates from pear trees in China with bleeding canker necrosis [9], but it was extended by a large number of strains isolated from monocot plants from Japan [10, 11]. *D. fangzhongdai* strains were associated with soft rot symptoms of many ornamental and economically important staple food plants [10, 12, 13], thereby highlighting the broad host range of the species.

While there is little information regarding associated economic damages and the extent of its occurrence in different host plants outside of Asia, Alič et al. [14] recently identified *D. fangzhongdai* as the causative agent of soft rot of orchids in commercial production in Europe, starting with material from Asia [11]. Moreover, as previously reported, bacteriophages of different families, and active against *D. fangzhongdai*, were isolated from a wastewater treatment plant not associated to the orchid production site. This would suggest that *D. fangzhongdai* bacteria may be more widespread in nature than could currently be concluded on the basis of symptoms in plants. Its occurrence in water would suggest that it may potentially have a wider ecological niche than genomically close *Dickeya* spp., that is, *Dickeya dadantii*, *Dickeya dianthicola*, and *D. solani*.

Previous experience with *D. solani* has shown that novel species or isolates can lead to clonal spread and high losses in affected host plants [15]. Together with repeated introductions of *D. fangzhongdai*, the co-occurrence of genetically and phenotypically diverse strains on the same plants (e.g., B16 and S1 on orchids, as reported by Alič et al. [11]) increases the probability of the development of recombined strains with novel pathogenic potential and may present a risk to agriculturally important plants. Their aggressiveness, high maceration potential on various plant tissues, and persistence in potato plants further exacerbate the risk for agriculture.

Therefore, in this paper, we analysed the genomic characteristics of the *D. fangzhongdai* species, compared it to the other *Dickeya* species and determine the inter- and intra- species diversity. The study addressed the question whether the presence of the isolates in a specific environment is associated to a specific set of genes (water vs plant symptoms, monocots vs dicots, different geographical origin). We also analysed the virulence gene arsenal, in order to evaluate the virulence potential of this species.

## Methods

### *Dickeya* strain selection

All *D. fangzhongdai* genomes publicly available in the NCBI database were included in this study. These genomes were compared to five *D. solani*, four *D. dadantii*, five *D. dianthicola*, five *D. chrysanthemi*, seven *D. zeae*, one *D. aquatica*, two *D. paradisiaca* and two unassigned *Dickeya* genomes extracted from the NCBI database. Information on the provenance and genomic data of the *D. fangzhongdai* strains used in this study are summarized in Table 1. The accession numbers and phylogenetic position of the other *Dickeya* strains used for the SiLix analyses are presented in Additional file 1: Figure S1.

**Table 1:** General genomic features of the different *Dickeya fangzhongdai* genomes

D. fangzhongdai strain	Strain origin	Source of isolation/host	Year of isolation	Sequencing technology	Accessions	Genome size (bp)	No. of contigs	No. of scaffolds	GC ratio (%)	Predicted PEGs	Predicted RNAs
JS5^**T**^	China	Pear tree	2009	Illumina HiSeq	CP025003	5,027,163	–	1	56.8	4478	98
B16	Slovenia	*Phalaenopsis* orchid	2010	Illimina and Ion Torrent	JXBN00000000	4,843,044	53	–	56.8	4603	56
S1	Slovenia	*Phalaenopsis* orchid	2012	Illimina and Ion Torrent	JXBO00000000	4,962,950	51	–	56.9	4738	74
MK7	Scotland (UK)	River water	–	454	AOOO01000000	4,905,506	66	21	56.6	4648	67
NCPPB 3274	St. Lucia	Aglaonema	1983	454	AOOH01000000	5,115,016	62	15	56.5	4838	82
M005	Malaya	Waterfall	2013	Illumina MiSeq	JSXD00000000	5,105,736	138	–	56.6	4784	73
M074	Malaya	Waterfall	2013	Illumina MiSeq	JRWY00000000	4,952,363	145	–	56.8	4630	71
ND14b	Malaya	Waterfall	2013	PacBio RSII	CP009460	5,052,868	1	–	56.9	4634	97

### Genome sequencing and assembly

The *D. fangzhongdai* B16 and S1 draft genomes assembled from Ion Torrent sequencing data [16] exhibited poor quality. Therefore, both genome sequences were improved by Illumina sequencing performed by Viroscan 3D (Lyon University, Faculté de Médecine et de Pharmacie, 69,008 Lyon). Paired-end 2 × 150 bp sequencing was conducted on an Illumina NextSeq500 instrument, with a High Output 150-cycle kit. CLC Genomics Workbench (Version 9.5.2, Qiagen Bioinformatics) was used to assemble 9,529,152 (mean length 149 bp) and 9,834,144 reads (mean length 149 bp) for strains S1 and B16, respectively. Final sequencing coverages were 143x and 150x with 51 and 53 scaffolds for strains S1 and B16, respectively.

### Annotation and comparison of the *Dickeya* genomes

The functional annotation of predicted genes was achieved using Rapid Annotation Subsystem Technology (RAST) server (http://rast.nmpdr.org/rast.cgi, Aziz et al. [17]) with the Glimmer 3 gene caller [18]. Genes of interest (Tables 3, S1, S2 and S3) were manually annotated and assigned to functional classes with the help of the Psi-Blast server.

Average nucleotide identity (ANI) was computed using the JSpecies package version 1.2.1 with the MUMer algorithm (http://jspecies.ribohost.com/jspeciesws/). In silico DNA-DNA Hybridization (DDH) was calculated according to [19], using a dedicated pipeline (http://ggdc.dsmz.de/).

The pan-genome (the entire set of gene families found in genomes of a species), core genome (set of gene families shared by all strains of a species), and species-specific genes (genes unique to one species) were determined using a homology constraint of 80% amino acid identity and 80% alignment coverage. Two R scripts were used to calculate rarefaction (core genome) and accumulation (pan genome) curves, as described by Meric et al. [20].

As we were working with draft genomes, some split or truncated genes were noted in the final genome assembly. Additional analysis of the annotated draft genomes was conducted to eliminate sequencing errors for the genes of interest (virulence genes, secondary metabolite pathways, specific genes). Presence and position of those genes was manually inspected to detect split genes, truncated genes at the end of contigs and missing genes.

Visualization and comparison of genomes was conducted on the CGView server (Circular Genome Viewer, https://server.gview.ca/) with the BlastAtlas tool [21]. Two complete genomes (ND14b and JS5) were used as reference genomes. Further, genome-to-genome comparison was conducted using bi-directional protein-protein BLAST sequence comparison of translated open reading frames (ORFs) with a 10^− 5^ e-value threshold. Genes were considered as strain-specific if the identity of the encoded protein was lower than 80% of the full-length amino acids sequence of the longest protein.

### Phylogenetic analysis

In silico multilocus sequence analysis (MLSA) was performed on 1162 concatenated amino acid orthologous sequences. Clustering of orthologous sequences into homologous families was conducted using the SiLix software package [22], using an 80% identity threshold on full-length proteins. Orthologous sequences were aligned with the Muscle software [23], then concatenated, and alignments were curated using Gblocks [24]. Phylogeny was performed using the PhyML algorithm [25] with the following settings: substitution model LG, 100 bootstraps, model-given amino acid equilibrium frequencies, invariable sites optimized, tree searching NNI, starting tree BioNJ. *Pectobacterium atrosepticum* was used as an outgroup. The iTOL tool was used to visualize the phylogenetic trees [26].

### Identification of mobile and extrachromosomal elements

The prophage identification tool PHAge Search Tool – Enhanced Release (PHASTER) was used to determine the region containing prophage-like elements in bacterial genomes (http://phaster.ca/) [27]). Prediction of clustered regularly interspaced short palindromic repeats (CRISPRs) was done using CRISPRfinder (http://crispr.i2bc.paris-saclay.fr/Server/) [28]. Insertion sequences and genomic islands were identified using the ISfinder (https://isfinder.biotoul.fr/general_information.php) [29] and IslandViewer 4 (http://www.pathogenomics.sfu.ca/islandviewer/) [30], respectively.

### Presence of virulence-associated genes

The presence of *Dickeya* virulence-associated genes previously reported by Reverchon and Nasser [4] was assessed using BLASTp analysis. The *D. fangzhongdai* core genome was compared to *D. dadantii* 3937genome annotation from the ASAP database [31] to determine the presence of known virulence determinants and regulators. Furthermore, genomic regions containing secondary metabolite biosynthesis gene cluster were identified using AntiSMASH software (version 4.1.0, 10.1093/nar/gkv437) [32].

### Functional classification of the specific genes

*D. fangzhongdai* species-specific gene families i. e. families absent in or sharing below 80% homology with the gene families present in other *Dickeya* species, were extracted from the SiLix output.

The COG categorization of species-specific genes was conducted by submitting predicted CDS to the EggNOG 4.5 database [33] and subsequent extraction of COG categories. Furthermore, signatures for protein families, domains and repeats was determined using Psi-Blast [34]. The predictions were performed on the basis of *D. fangzhongdai* ND14b or JS5 gene sequences. The manual functional classification of the species-specific genes was conducted on the basis of RAST annotation, COG categorisation, Psi-blast and structure signatures information.

### Antibiotic resistance

Susceptibility to antibiotic streptomycin was tested for *D. fangzhongdai* strains S1, B16, MK7, JS5, and NCPPB 3274. The bacteria were grown on Luria Bertani (LB) medium with 1.5% agar. Bacterial suspensions were prepared in 10 mM PB buffer (1.07 g Na_2_HPO_4_, 0.4 g NaH_2_PO_4_·2H_2_O per liter of water, pH 7.2) from overnight cultures to the final concentration of 10^6^ cfu/mL. Antibiotic resistance was tested by spot plating 15 μL of bacterial suspensions to LB medium plates containing streptomycin (ranging from 5 to 100 μg/ml). Inoculated plates were incubated overnight at 28 °C.

## Results

### Statistics and features of the genomes

**Fig. 1:**
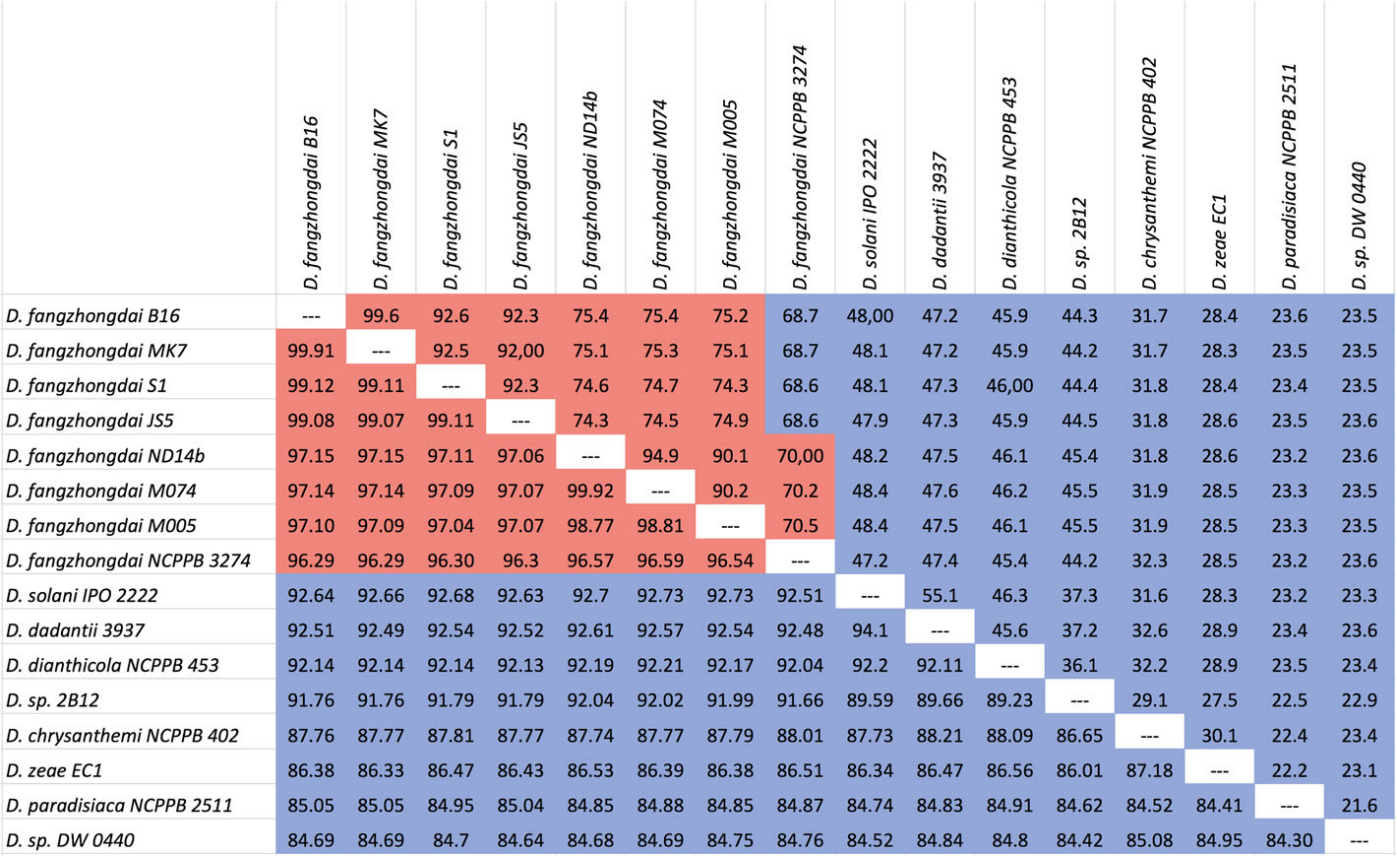
Definition of the *Dickeya fangzhongdai* species in silico DNA-DNA hybridization (DDH, upper triangle) and Average Nucleotide Identity (ANI, lower triangle) values of *Dickeya fangzhongdai* strains and representative strains of the other described *Dickeya* species. Strains belonging to the same species are highlighted in red. The specific threshold value is 96% for ANI and 70% for DDH

**Fig. 2:**
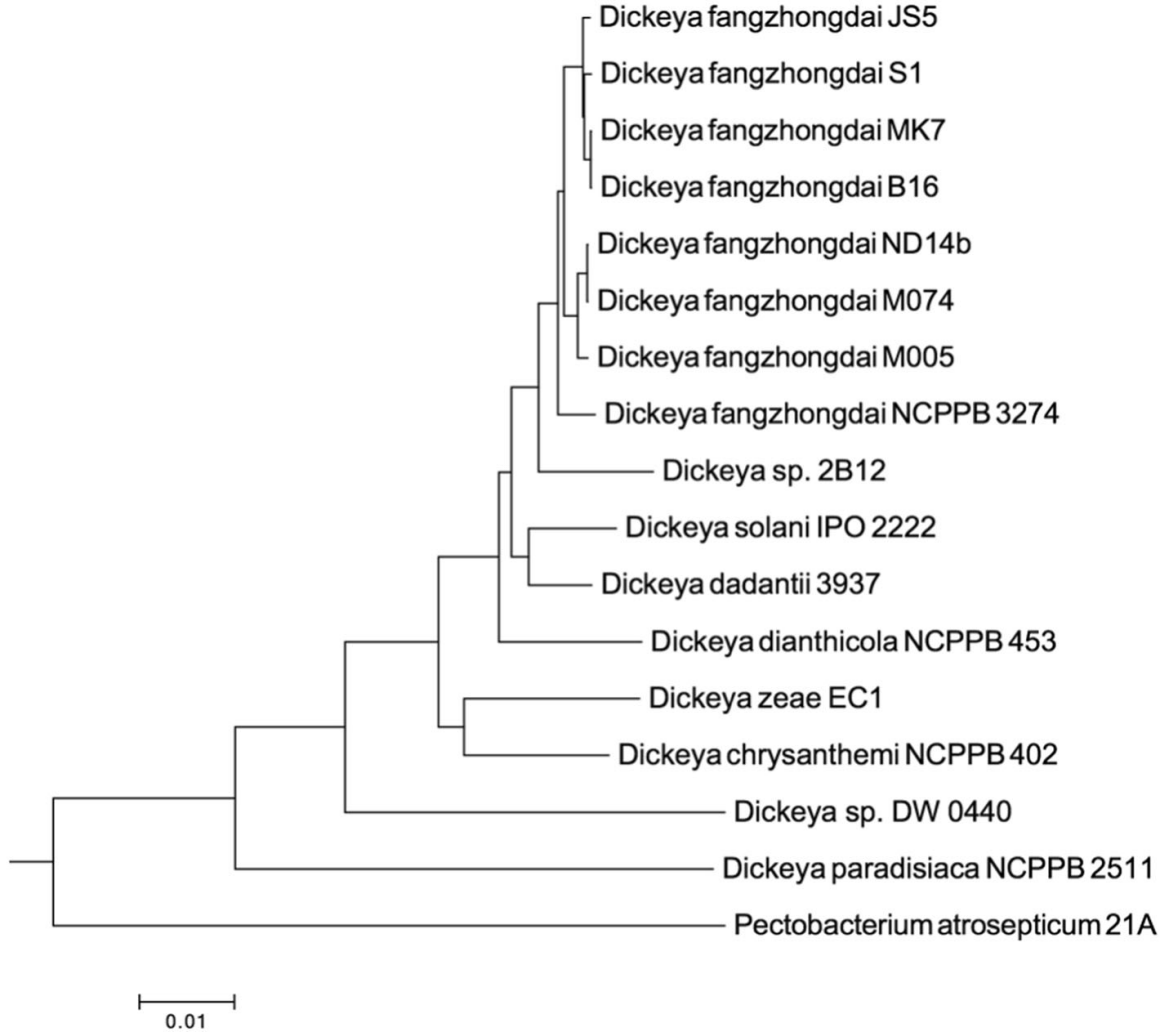
Phylogenetic tree of *Dickeya fangzhongdai* strains and representative strains of other described *Dickeya* species. The tree was constructed from concatenated sequences of 1162 homologous amino acid sequences (71,912 variable sites). One hunderd bootstrap replicates were conducted to assess the statistical support of each node. Bootstrap support values are 100% for all nodes but one. *Pectobacterium atrosepticum* 21A was used as an outgroup

This study included the eight genomes of *D. fangzhongdai* that were available in GenBank databases. The Nd14b genome has been completely sequenced using PacBio technology [35]. We built improved versions of B16 and S1 strains genomes using Illumina technology, which resulted in the availability of seven draft genomes that comprise approximately 1–150 contigs, precluding synteny studies of the *D. fangzhongdai* species (Table 1). ANI and DDH analyses confirmed the assignation of the eight strains into the same species and their closeness to the *D. solani/D. dadantii/D. dianthicola* clade (Fig.1). This is consistent with the MLSA phylogenetic analysis (Fig. 2).

As already shown by DNA-DNA hybridizations [14], the NCPPB 3274 strain is at the limit of being part of the *D. fangzhongdai* species. Indeed, if the ANI values exceeded 96% threshold, the DDH values range from 68.6–70.5%.

### *D. fangzhongdai* core and accessory genomes

**Fig. 3:**
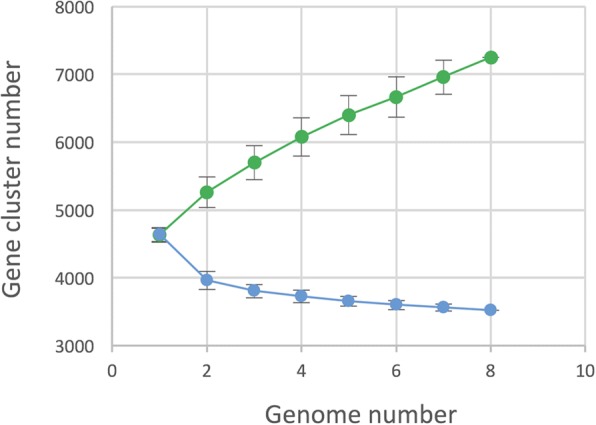
Definition of *Dickeya fangzhongdai* core and pan-genomes. Rarefaction (blue, core genome) and accumulation (green, pan genome) curves are presented. Randomized genome sampling was performed 100 times to obtain the average number of genes and the standard deviations for each added genome

To determine the species core and pan genomes, we conducted a comparative genomic analysis of *D. fangzhongdai* genomes annotated by the RAST platform, using the SiLix gene family clustering tool. Proteins were classified as homologous to another in a given family if the amino acid identity were above 80% on the full-length amino acid sequence. The core genome of *D. fangzhongdai*, defined as gene families shared by all strains of the species, comprises 3520 gene families and represents roughly three-quarters of all predicted encoding proteins in each strain. This core genome is highly conserved, since 86% of the core genome genes share at least 95% identity. The species pan-genome, defined as the entire set of gene families found in the genomes of the species, includes 7249 gene families that represent twice the number of the core genome genes. This continuously increases with the addition of each genome, thereby indicating that the *D. fangzhongdai* pan-genome is still far from closeness (Fig. 3).

### Most known *Dickeya* virulence determinants are present in *D. fangzhongdai*

Several virulence factors have been characterized in the *D. dadantii* 3937 model strain. Soft rot symptoms are caused by the production and secretion of plant cell wall degrading enzymes, pectinases and cellulases secreted by the Out T2SS (type 2 secretion system) and proteases secreted by the Prt T1SS. Furthermore, an efficient colonization of the host plant requires the production of several additional bacterial factors that are either involved in the adhesion and penetration into plant tissues or in the adaptation to the different stresses encountered by the bacteria inside plants. These factors include cell envelope components (LPS and EPS), motility, efficient iron uptake systems, as well as defences against acidic and oxidative stresses or against antibacterial compounds produced by the plant [4]. All *D. fangzhongdai* strains possess the entire battery of plant cell wall degrading enzymes present in *D. dadantii* 3937 and their cognate secretion systems; they also harbour the second Stt T2SS system that is present in *D. dadantii* but is not widely spread among the different *Dickeya* species. The *D. fangzhongdai* core genome also includes the vast majority of the other bacterial factors shown to be involved in interactions with plants in *D. dadantii* [4], with two exceptions: the gene encoding the acid shock periplasmic Asr protein that plays a role in survival under acid conditions is missing in all *D. fangzhongdai* strains as do the *iaaMH* genes involved in auxin production. However, the presence of these genes in *Dickeya* genomes varied within the genus.

#### Diversity in other protein secretion systems

Apart from the T1SS and T2SS systems, all *D. fangzhongdai* strains possess a T3SS and share the same repertoire of T3SS effectors. More diversity was observed for the T4SS, T5SS and T6SS.

Two types of T4SS might be present in bacteria, either associated with plasmid conjugation and DNA uptake/release or to protein secretion [36]. *D. dadantii* 3937 encodes both types of T4SS, a VirD2/VirD4/Trb locus present in an integrative conjugative transposon element (ICE) and another one regrouping only a *virB* operon [37]. Conserved VirD2/VirD4/Trb systems are present in *D. fangzhongdai* ND14b, M074 and M005. The M005 locus is highly syntenic with *D. dadantii* 3937, while in the two other strains it is present in a different common genetic context. In addition, like 3937, ND14b, M074 and M005 possess a *virB* operon. A *virB* operon is also present in *D. fangzhongdai* S1, and in two copies in *D. fangzhongdai* NCPPB 3274. No *virD4* or *virD2* genes are present in these two strains, however noticeably, in certain bacteria like *Bordetella pertussis*, a T4SS was shown to be functional for protein secretion even in the absence of a VirD4 homolog [38]. Finally, *D. fangzhongdai* strains MK7 and B16 did not contain any genes connected to T4SS, and JS5 possesses only a *virD4*-related gene that is located near a *pil-tra* gene cluster that is predicted to be involved in plasmid conjugative transfer. Therefore, among the eight analysed *D. fangzhongdai* strains, only five harbour T4SS and three of them additionally possess a module involved in conjugation.

Type V (T5SS) and type VI (T6SS) secretion systems are both involved in contact-dependent inter- and intra-species competition systems. T5SS are two-partner secretion systems called Hec, Tsp or Cdi (contact-dependent inhibition) that consist of an outer membrane TspB/HecB protein allowing the secretion of a large TspA/HecA multidomain protein. TspA/HecA protein comprises an N-terminal transport domain, a large hemagglutinin-like region that is proposed to form a fibre-like structure and typically a C-terminal toxin domain beginning with the VENN motif [39].

**Fig. 4:**
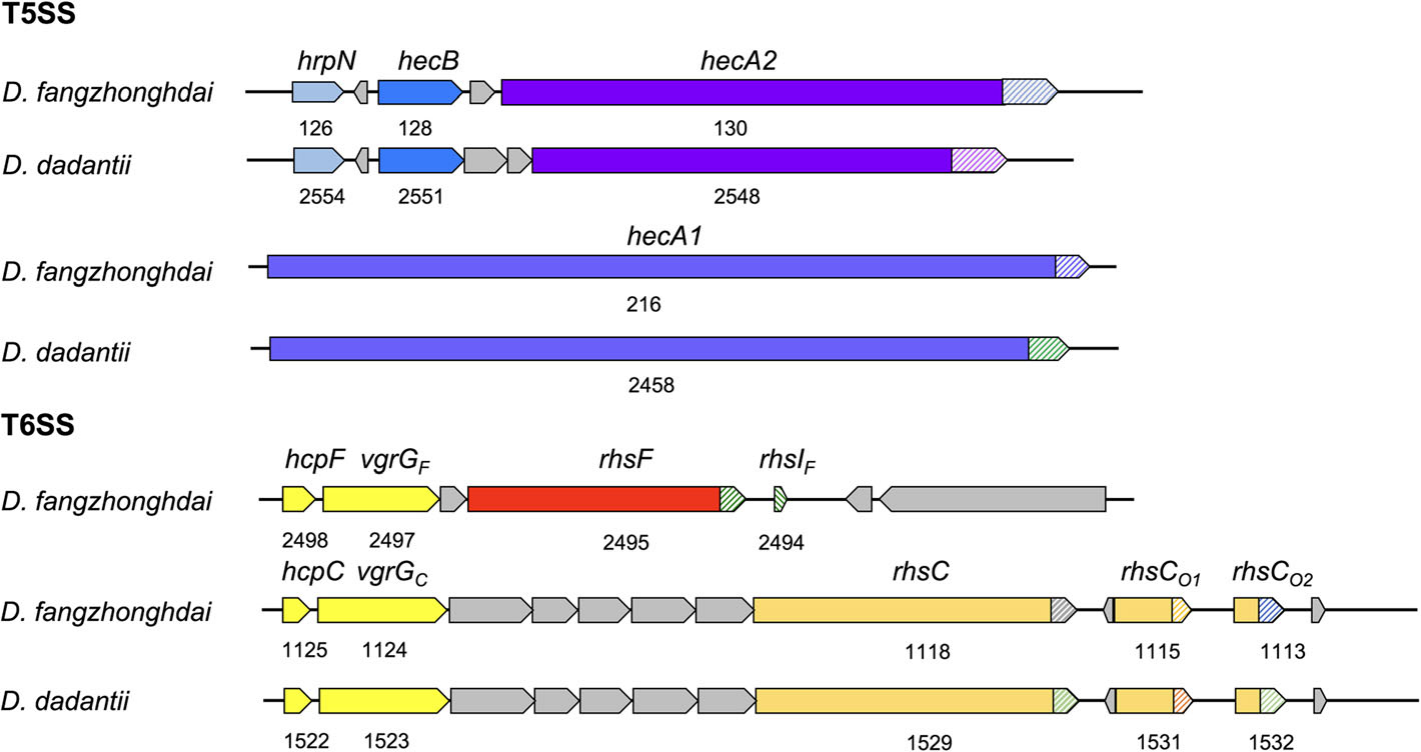
Genes or genetic clusters of T5SS and T6SS-related toxin/antitoxin systems in the model *Dickeya fangzhongdai* strain ND14b. The different clusters were compared to the corresponding clusters present in the 3937 *Dickeya dadantii* model strain. The *rhsF* cluster is found only in the *Dickeya fangzhongdai* species even if it is highly similar to the *hcp-vgrG-rhsB*_3937_ cluster (see text for details)

*D. dadantii* 3937 harbours two T5SS systems that have been shown to act in contact-dependent growth inhibition [39, 40]. In Nd14 and JS5, the only *D. fangzhongdai* complete genomes available, one *hecB* and two *hecA* genes, were identified in regions showing high synteny with the related *D. dadantii* 3937 regions. In ND14b, both HecA proteins are 59 and 63% identical (76 and 77% similar) to their counterparts in 3937; the C-terminal toxin portion is different in these four proteins (Fig. 4). The JS5 HecA proteins are even more similar to the 3937 proteins (66 and 73% identical, 84–87% similar). All other *D. fangzhongdai* strains possess a *hecB* homolog. The detection of HecA encoding genes is ambiguous since the reads assembly is often interrupted in these very long (up to 4000 amino acids) modular proteins that contain a high number of repeats. Most of the genes are truncated at the end of contigs leading to truncated predicted proteins. Nevertheless, indications of the presence of *hecA1* and h*ecA2* genes were found in all *D. fangzhongdai* but the MK7 genome (Additional file 2: Table S1).

T6SS systems are contractile nanomachines that function in a manner analogous to an inverted phage tail and tube to deliver effectors in target cells. They comprise the secretion machinery encoded by the *imp/vas* operon, the hemolysin-coregulated protein (Hcp) and valine-glycine repeat protein G (VgrG) secreted proteins that form a membrane puncturing device, and effectors such as so called Rearrangement HotSpot (Rhs) proteins. The Rhs proteins are large composite proteins consisting of a large N-domain that contain YD-peptide repeats and a highly variable C-domain that harbours toxic activity. To block the toxic activity of the Rhs protein, a small immunity protein RhsI is produced, whose encoding gene is very often located just adjacent to the corresponding rhs gene [40]. The *rhs* genes are very often located close to *hcp-vgrG* genes. *D. dadantii* 3937 possess three copies of these *hcp-vgrG-rhs* clusters and one of them is accompanied by a number of additional orphan Rhs-CT/RhsI pairs. All *D. fangzhongdai* strains possess a well-conserved entire *imp/vas* operon. The *Hcp-vgrG-rhsC*_3937_ cluster is largely conserved in all strains, even if genome assembly problems clouded the identification of some of these genes (Additional file 2: Table S1). In ND14b, the N-end of the RhsC and the orphan-related proteins are highly similar to their 3937 counterparts; however, the CT regions are different (Fig. 4). On the contrary, the *hcp-vgrG-rhsB*_3937_ cluster was identified only in *D. fangzhongdai* NCPPB3274 and S1. We did not detect any homolog to the *hcp-vgrG-rhsA*_3937_ cluster in the same genomic location in *D. fangzhongdai*; however, interestingly, all *D. fangzhongdai* strains possess another *hcp-vgrG-rhs* cluster named *hcp-vgrG-rhsF* in which the RhsF protein is highly similar to the *hcp-vgrG-rhsB*_3937_ (88% identity/95% similarity even in the CT region) (Fig. 4).

#### Secondary metabolites and corresponding pathways

Analysis with the antiSMASH server [32] identified seven secondary metabolite biogenesis clusters in the *D. fangzhongdai* core genome, as summarized in Table 2. Three of these clusters encode the biosynthesis of well-known secondary metabolites produced by all *Dickeya* analysed so far. The chrysobactin and achromobactin clusters govern the biosynthesis of the two siderophores responsible for the efficient uptake of iron and are involved in bacterial survival and virulence [4]. The *ind-vfm-expI* cluster groups genes that are involved in the synthesis of the antioxidant indigoidine molecule as well as in the quorum sensing regulation mediated by an AHL and the new VFM signal [2, 41]; the production of indigoidine by *D. fangzhongdai* was experimentally confirmed in previous studies [11]. All *D. fangzhongdai* strains also possess the gene cluster involved in the biosynthesis of the zeamine toxin. Two other secondary metabolite-related clusters were predicted to be involved in the biosynthesis of either cyanobactin-related or thiopeptide-related molecules. Cyanobactins are small cyclic peptides with variable functions, the most common activity is cytotoxic [42]. Several thiopeptides present an antibiotic activity [43]. However, we could not connect these clusters to any known molecule. The last cluster common to all *D. fangzhongdai* strains is encoding a non-ribosomal peptide synthase (NRPS) and polyketide synthases (PKS) complex. These NRPS/PKS complexes are involved in the synthesis of polymers of peptidyl/carbonyl chains. In this cluster, Nd14b possesses an additional and rather long NRPS encoding gene that indicates the production of slightly different polymers than in other *D. fangzhongdai* strains.

**Table 2:** Secondary metabolite gene clusters identified with AntiSMASH in *Dickeya fangzhongdai* genomes

	RAST IDs	D. fangzhongdai strains							
		JS5	S1	B16	MK7	ND14b	M074	M005	NCPPB3274
Chrysobactin	4020-4056^a^	+	+	+	+	+	+	+	+
Achromobactin	917-929^a^	+	+	+	+	+	+	+/−^c^	+
*ind-vfm-expIR*	2414-2478^a^	+	+	+	+	+	+	+	+
Zeamine	1047-1086^a^	+	+	+	+	+	+	+	+
Cyanobactin	3008-3027^a^	+	+	+	+	+	+	+	+
Thiopeptide	584-595^a^	+	+	+	+	+	+	+	+
transAT PKS/NRPS	4466-4509^a^	+	+	+	+	+	+	+	+
Arylpolyene	2584-2618^a^	–	+	–	–	+	+	+	+
Bacteriocin	1483-1510^a^	–	+	+	+	+	+	+	+
Nrps	4516-4553^b^	+	+	+	+	–	–	–	(2)^d^

^a^genes based on the ND14b Rast ID

^b^genes based on the JS5 Rast ID

^c^genome assembly problems for this region in M005

^d^NCPPB3274 harbours two additional NRPS clusters, different from the cluster found in JS5, S1, B16 and MK7

+, presence of the genetic cluster; +/−, partial gene cluster; −, absence of the genetic cluster

In addition to the seven clusters described above, shared by all *D. fangzhongdai*, further five clusters detected by AntiSMASH were found only in some *D. fangzhongdai* strains—two of them were predicted to comprise genes involved in the synthesis of a bacteriocin or the biosynthesis of arylpropylene. The bacteriocin synthesis cluster is present in all *D. fangzhongdai* strains, except JS5. The arylpoliene biosynthesis cluster was identified in all *D. fangzhongdai* strains, excluding MK7, JS5 and B16 isolates. The third cluster encodes an NRPS cluster that is shared by the JS5, B16, S1 and MK7 strains. Since no homologous genetic cluster could be identified in any other bacterial genome in databases, no information on the produced secondary metabolite is available. NCPPB3274 harbours two additional NRPS clusters. One of them encodes a trans-acyltransferase polyketide synthase cluster that presents the same genetic organization as the oocydin A clusters found in S*erratia plymuthica* and S*erratia marcescens* strains.

#### Virulence regulatory pathways

*D. fangzhongdai* species showed homology to *D. dadantii* 3937 virulence genes, and only minor differences were noted in their content. This is also the case for the regulatory proteins regulating the virulence programme. Indeed, all the regulators identified in *D. dadantii 3937* as controlling virulence factors [4] were present in the core genome of the *D. fangzhongdai* species, including global regulators, nucleotide-associated proteins and post-transcriptional regulation molecules.

### Species-specific genes might be important for species virulence

To extract unique features of the *D. fangzhongdai* species, a genome comparison was conducted by comparing the *D. fangzhongdai* core genome with the genomes of 31 strains belonging to the seven other *Dickeya* species (marked on the MLSA tree, in Fig. S1). Species-specific genes are defined as present in all *D. fangzhongdai* strains and absent in all other *Dickeya* species (using a specific threshold of 80% identity on the full-length protein sequence). Only 38 gene families specific to *D. fangzhongdai* species were detected, thereby confirming the high genetic conservation of the *Dickeya* genus. Approximately one-half of these genes encode hypothetical proteins (19 proteins with 9 smaller than 60 amino acids). These species-specific genes were not clustered together in a region of the genome; rather, they were evenly scattered throughout the genome. Table 3 presents the characteristics of the 25 *D. fangzhongdai*-specific genes for which a function is predicted or genes that have protein motives and/or genes that were found in other bacteria genera. A significant proportion of the species-specific genes with predicted function are genes involved in regulation (four genes) and different types of metabolism (six genes). Another species-specific gene encodes a short orphan NRPS protein (512 AA) that did not correspond to any known secondary metabolite biosynthesis pathway.

**Table 3:** *Dickeya fangzhongdai* species-specific genes^a^

RAST ID	Size (AA)	Annotationa^b^	Domains/Motives^c^	Functional classification	COG^d^	COG class^d^	KEGG^d^	Gene conserved in^e^
Dickeya_fangzhongdai_ND14b.0429	457	Pectate lyase	Pec lyase superfamily	protein secretion	–	–	–	–
Dickeya_fangzhongdai_ND14b.0753	131	Ketosteroid isomerase-related protein	NTF2 like superfamily	metabolism	–	–	–	*Comamonas testosteroni*
Dickeya_fangzhongdai_ND14b.0754	212	Thioredoxin-like protein clustered with PA0057	Thioredoxin like superfamily	Posttranslational modification	–	–	–	*Comamonas testosteroni*
Dickeya_fangzhongdai_ND14b.0755	323	Nitrogen assimilation regulatory protein Nac	HTH superfamily, Periplasmic binding protein Type 2 superfamily	regulation	–	–	–	*Polaromonas* sp. CF318
Dickeya_fangzhongdai_ND14b.0976	317	Transcriptional regulator containing an amidase domain and an AraC-type DNA-binding HTH domain	GAT_1 superfamily, HTH_AraC superfamily	regulation	–	–	–	*Burkholderia lata*
Dickeya_fangzhongdai_ND14b.1018	556	Pyruvate decarboxylase (EC 4.1.1.1); Alpha-keto-acid decarboxylase (EC 4.1.1.-)	TPP_enzyme_PYR, TPP_enzyme_M, TPP_enzyme_C superfamily	metabolism	COG3961	GH	K04103	*Pectobacterium carotovorum*
Dickeya_fangzhongdai_ND14b.1127	562	Chaperone protein hscC (Hsc62)	HSP70 superfamily, NBD_sugar kinase/HSP70/actin superfamily	Others	COG443	O	K04043,K04045	*Pectobacterium carotovorum*
Dickeya_fangzhongdai_ND14b.1128	891	Uncharacterized J domain-containing protein YbeS, predicted chaperone	DnaJ superfamily, DUF805 supefamily	Others	–	–	–	–
Dickeya_fangzhongdai_ND14b.1129	237	Glycosiltransferase family protein	DUF1266 superfamily	undefined	–	–	–	*Pectobacterium carotovorum*
Dickeya_fangzhongdai_ND14b.1510	92	MarR family transcriptional regulator	HTH superfamily	regulation	COG1733	–	–	*Burkholderia* sp. H160
Dickeya_fangzhongdai_ND14b.1511	78	Hypothetical transmembrane protein	–	hypothetical protein	COG2259	S	–	*Xanthomonas arboricola*
Dickeya_fangzhongdai_ND14b.1828	407	Hypothetical protein	DUF1501 superfamily	hypothetical protein	COG4102	S	–	–
Dickeya_fangzhongdai_ND14b.1829	625	Glycosyltransferase	Glyco_transf_GTA_type suerfamily	metabolism of cell wall	COG1215	M	K00698	–
Dickeya_fangzhongdai_ND14b.2289	73	Molybdopterin-guanine dinucleotide biosynthesis protein MobB	p-loop NTPase superfamily	metabolism	COG1763	H	K03753	–
Dickeya_fangzhongdai_ND14b.2311	53	hypothetical protein	DUF1127 superfamily	hypothetical protein	–	–	–	–
Dickeya_fangzhongdai_ND14b.2427	227	hypothetical protein	–	hypothetical protein	–	–	–	*Pectobacterium carotovorum*
Dickeya_fangzhongdai_ND14b.2499	458	Permeases of the major facilitator superfamily	MPS superfamily	transport	–	–	K18326	*Erwinia toletana*
Dickeya_fangzhongdai_ND14b.2993	96	hypothetical protein	DUF2631 superfamily, PilP superfamily	hypothetical protein	–	–	K12291	*Serratia* sp. ATCC 39006
Dickeya_fangzhongdai_ND14b.3123	511	Siderophore biosynthesis non-ribosomal peptide synthetase modules	Adenylate forming domain, Class I superfamily	metabolism of secondary metabolites	COG1020	Q	–	*Serratia* sp. ATCC 39006
Dickeya_fangzhongdai_ND14b.3971	158	GNAT family N-acetyltransferase	RimI domain, NAT_SF superfamily	Others	–	–	–	–
Dickeya_fangzhongdai_ND14b.3994	326	Quinone oxidoreductase (EC 1.6.5.5)	MDR superfamily	metabolism	COG0604	C	K00344	Rahnella aquatilis
Dickeya_fangzhongdai_ND14b.3997	314	Transcriptional regulator, LysR family	HTH superfamily, Periplasmic binding protein Type 2 superfamily	regulation	–	–	–	Pantoea sp. AS-PWVM4
Dickeya_fangzhongdai_ND14b.4503	105	Quaternary ammonium compound-resistance protein SugE	EamA superfamily	transport	COG2076	P	K03297,K11741	Cronobacter
Dickeya_fangzhongdai_ND14b.4575	149	Hypothetical protein	DUF1311 superfamily	hypothetical protein	–	–	–	–
Dickeya_fangzhongdai_ND14b.4576	737	Putative cytoplasmic protein	DUF4764 superfamily, Pesticin superfamily	undefined	–	–	–	–

^a^The table presents species-specific genes that have protein motives and/or were found in other bacteria genera. Thirteen hypothetical proteins without any predicted motives and without any similar proteins in other bacteria were omitted from the Table

^b^Manually curated RAST annotation

^c^Proteins without conserved domain or motives are marked with -

^d^Proteins without identified COG or KEGG orthologes are marked with -

^e^Treshold for conservation designation is 70% identity over 70% sequence length

Interestingly, *D. fangzhongdai* contains an additional and unique pectate lyase gene. The encoding protein differs significantly from the pectate lyase found in other bacterial plant pathogens or in other *Dickeya* spp., except in *D. chrysantemi* Ech 1591 (74% protein sequence identity). Based on protein structure prediction, this protein belongs to family 10 of polysaccharide lyase (PL10). A signal peptide was identified to be 27 AA long, thereby indicating extracellular or periplasmic localisation of the protein. Since PL10 from *D. chrysanthemi* Ech 1591 has not been characterised yet, no other properties of the protein are available.

### Genomic diversity in the *D. fangzhongdai* strains

#### D. Fangzhongdai clades

As visualized in the MLSA phylogenetic tree (Fig.2), ANI/DDH values clearly indicate additional clustering within the *D. fangzhongdai* species. Indeed, the strains can be divided into three branches—a JS5-like and a ND14b-like cluster and the more divergent NCPPB3274 strain (Fig. 1). For both clusters, the genome conservation between the members of the same cluster is extremely high. Indeed, strains within the JS5-like and ND14b-like cluster shared on average of 85 and 88% of the gene families, respectively; moreover, approximately 95% of these genes were at least 95% identical. Each clade shares a pool of 90–160 genes that are shared by the clade members but are absent from the other *D. fangzhongdai* strains. Interestingly, most of these genes were not present in other *Dickeya* genomes (Additional file 3: Tables S2 and S3). However, approximately one-half of these clade-shared genes mainly comprised short hypothetical proteins that were below 60 AA long (49 genes in the JS5-like and 61 genes in the ND14b-like clade). The remaining genes corresponded to (i) metabolism, including secondary metabolite biogenesis, (ii) transport, (iii) regulation or (iv) other functions (Additional file 3: Tables S2 and S3; Additional file 1: Figure S1). In contrast to species-specific genes, several of these genes shared by clade members are clustered in genomic regions (GR), and several of these clustered genes have a predicted function (Table 4). Three genomic regions were identified in the JS5-like clade. The GR1 gene content indicates an involvement in carbon metabolism. GR2 groups three very large proteins of unknown functions and a gene involved in transport. GR3 contains a NRPS biosynthesis cluster operon, as already described above.

**Table 4:** Genomic regions of the JS5 and ND14b genomic clades

Clade	Genomic region	Size of GR (nt)	Genes RAST ID	Presence of horizontal transfer signature	Predicted function	Region conserved in^c^
JS5-like clade^a^	GR1	7033	3204-3211^a^	No	metabolism (putative)	D. dianthicola RNS04.9
	GR2	11,303	3941-3945^a^	No	Transport (putative)	–
	GR3	12,717	4354-4357^a^	No	NRPS	–
ND14b-like clade^b^	GR4	4729	317-323^b^	No	metabolism (putative)	Leclercia sp. LSNIH3
	GR5	11,197	1736-1746^b^	Yes	ICE (partial)	D. dadantii 3937
	GR6	11,493	2589-2598^b^	No	metabolism (putative)	D. dadantii 3937
	GR7	9241	2906-2913^b^	No	metabolism (putative)	–
	GR8	4732	3899-3903^b^	No	type I pilus	–

^a^genes based on the JS5 Rast ID

^b^genes based on the ND14b Rast ID

^c^the best hit in BLASTn analysis

Genomic regions found in each of the JS5 and Nd14b genomic clades that are absent from the other *Dickeya fangzhongdai* genomes are presented. Conserved regions share at least 70% identity over 70% sequence length

Five genomic regions were identified in the ND14b-like clade. Three of them (GR4, 6 and 7) comprised mainly genes related to metabolism and transport. The GR7 sequence is not conserved in other bacteria. In contrast, homologs of GR4 and GR6 were found in *Leclercia* sp. LSNIH3 and *D. dadantii* 3937, respectively. GR5 comprises the second VirD2/VirD4/Trb T4SS system described above. Biologically the most interesting genomic region is GR8. It contains five genes that code for a sigma-fimbriae structure. No homologous region was found in other bacteria. However, based on the genetic structure of the region, it is related to the *csu*-like operon of *Pseudomonas aeruginosa*. CsuA/BABCDE pili normally comprise six proteins; however, only five related proteins were found in the ND14b-like clade. Therefore, protein sequences of the GR6 cluster were compared to the well-defined CsuA/BABCDE pili proteins from *Acidovorax baumannii* 19606 to identify the putative functions of the proteins in GR6. The GR6 proteins share 58–73% similarity with their *A. baumannii* strain 19606 counterparts, namely CsuE (gene ID 3899), CsuD (gene ID 3900), CsuC (gene ID 3901) and CsuA/B (ID 3903), predicted as tip adhesin, chaperone, usher protein and major pili subunit, respectively. However, one of the minor tip subunits CsuA or CsuB is missing in the ND14b-clade GR6. All the ND14b-clade corresponding proteins were predicted as non-cytoplasmic (TMHMM), although only the protein sequences encoded by the ID3902 and ID3899 genes contained a signal peptide, as predicted by SignalP4.1 (http://www.cbs.dtu.dk/services/SignalP/) [44] and Phobius (http://phobius.sbc.su.se/) [45] programs. Furthermore, the protein sequences encoded by the ID3902 and ID3901 genes possessed a predicted transmembrane region like the *A. baumannii* CsuA and CsuC proteins, respectively. A transmembrane region was also identified in the protein sequence 3900, although the corresponding CusD protein contains a signal peptide rather than a transmembrane sequence in the same region (TMHMM, SignalIP4.1). Based on our results, members of the Nd14b-like clade have genetic dispositions for type I like pili expression; however, functional pili have to be experimentally confirmed. The JS5-like clade members did not contain a *csuA/BABCDE* operon; however, few very short fragments of the pili proteins (below 100 AA) were conserved. For *Acinetobacter baumannii* 19606, it was shown that the *csu* locus is involved in bacteria attachment and biofilm formation on abiotic surfaces [46].

#### *D. fangzhongdai* diversity resides mainly in genes of extrachromosomal origin

**Fig. 5:**
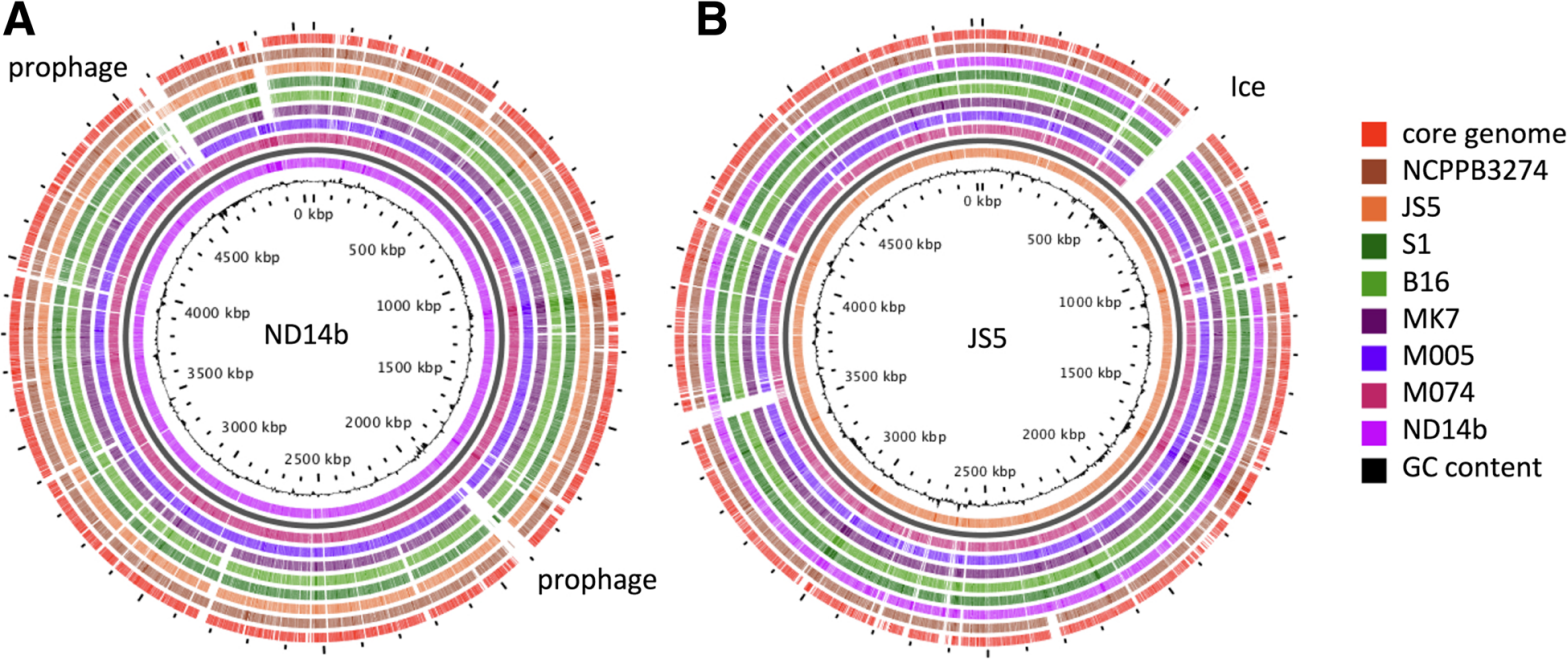
Genome BLAST atlas of *Dickeya fangzhongdai* strains. A: ND14b is the reference genome. B: JS5 is the reference genome. Genes belonging to the core genome are mapped (outmost red circle). Regions corresponding to prophages or ICE are marked

To further analyse the diversity between *D. fangzhongdai* stains, we performed a Blast-Atlas analysis based on the complete genomes of one representative of each clade, ND14b and JS5 (Fig. 5). This revealed that, in both clades, the conserved genes are evenly spread over the entire genome and that M074 is very close to ND14b. Only a few genomic regions of the Nd14b/M074 and JS5 genomes are not present in any of the other *D. fangzhongdai* isolates. The two large Nd14b/M074 1.86–1.88 Mbp and 4.56–4.62 Mbp regions encode prophages. Four regions are present only in JS5. The 0.45 Mb and 4.1 Mb regions consist of genes encoding mobile elements and hypothetical proteins. The large 0.51–0.52 Mb region comprises 77 genes related to transposition, conjugative transfer systems and replication typical of Integrative and Conjugative Elements (ICE). The last region (0.9 Mb) groups genes that are either involved in metabolism or encoding mobile elements. The high accumulation of genes encoding extrachromosomal elements in our BLAST-Atlas analyses prompted us to identify such genes in each *D. fangzhongdai* genome (Table 5). The number of sequences related to mobile elements varies among the *D. fangzhongdai* isolates. The genomes were relatively poor with insertion sequences (IS), harbouring none (S1 genome) to up to five full IS elements per genome, as identified by the ISfinder tool. The elements were members of four different IS families, namely IS200/IS605, IS110, IS30, IS4 and IS3. Different combinations of elements were present but all genomes with IS elements contained an IS200/IS605 family (IS200 group). Most of the analysed *D. fangzhongdai* strains did not contain complete prophage sequences within their genomes, but only possess short prophage related fragments. Nevertheless, an intact prophage was found within the ND14b and M074 genomes and two different prophages in the NCPPB 3274 genome as detected by the Phaster server. One of the *D. fangzhongdai* NCPPB 3274 prophages corresponds to the one found in *D. dadantii* 3937, as 83% of the prophage region exhibited above 90% nucleotide identity. Sequences homologous to the second NCPPB 3274 prophage are present in *D. zeae* EC1 (89% of the prophage region exhibited above 90% nucleotide identity). Both prophages sequences corresponded to *Myoviridae* bacteriophages, namely to *Haemophilus* virus HP1 and HP2 and to *Enterobacteria* phage P88, respectively. CRISPR elements are very important features of bacterial genomes as they provide acquired immunity against viruses and plasmids [47]. All *D. fangzhongdai* genomes comprised multiple CRISPR sequences. The number of CRISPRs varies from four to seven complete CRISPRs among the isolates (CRISPRfinder tool), a variation also encountered in other *Dickeya* isolates and species. Furthermore, the S1 strain harbours an entire contig (contig 24) that presents all characteristics of a plasmid even if we did not succeed in closing it (see below). NCPPB 3274 also harbours a 92 Kb long contig that contains ten genes involved in conjugative transfer (related to IncF-*traBCNUHI* and a relaxase) and encoding a ParA plasmid partitioning protein. The other genes mainly encode hypothetical proteins and the contig sequence was not homolog to any bacterial or plasmid sequences. In the M005 strain, the IslandViewer server identified a large genomic island (ID_M005.4077 to 4134) that groups integrase genes as well as plasmid-like replication and recombination functions, as well as a conjugative machinery typical of ICEs related to the *Pseudomonas fluorescens* Pf-5 PFGI-1 genomic island [48]. Up to two other genetic clusters related to ICE elements, mainly to ICE elements found in other *Dickeya* spp. (ICEDda3937–1, ICEDdaEch586–1 and ICEDzeEch1591–1), were found in most *D. fangzhongdai* genomes, excluding the MK7 and B16 strains.

**Table 5:** Presence of different types of mobile elements and CRISPR in *Dickeya fangzhongdai* genomes

*Dickeya fangzhongdai* strain	No. of confirmed CRISPR elements	No. of intact prophages	No. of incomplete or questionable prophages	No. of full IS elements	IS families	
JS5T	5	–	1	3	IS200/IS605, IS110, IS3	
B16	7	–	8	3	IS200/IS605, IS30, IS3	
S1	3	–	6	0	–	
MK7	7	–	5	2	IS200/IS605, IS30	
NCPPB 3274	6	2	1	3	IS200/IS605, IS110, IS4	
M005	4	–	7	4	IS200/IS605, IS30	
M074	5	1	4	4	IS200/IS605, IS110, IS3	
ND14b	4	1	2	5	IS200/IS605, IS110, IS3	

### The strain S1 plasmid

**Fig. 6:**
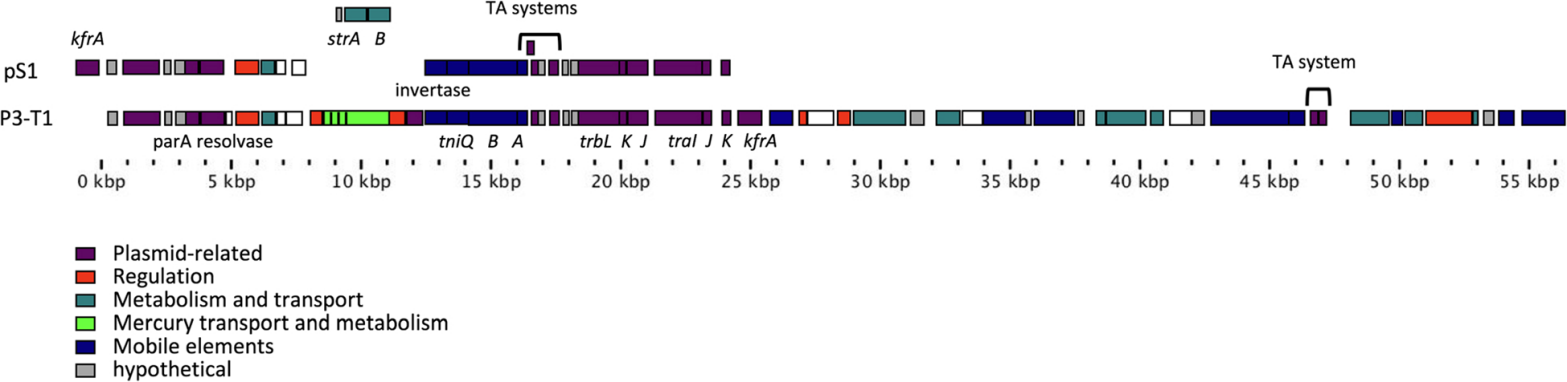
The pS1 plasmid. Gene content of the pS1 plasmid was compared to the *Acidovorax* sp. T1 pS3-T1 plasmid. Genes related to plasmid maintenance, replication and transfer (mauve boxes) are conserved between both plasmids

The 23 Kb-long S1 contig 24 regroups several plasmid-related proteins involved in replication (gene ID2790), plasmid stabilization (genes ID2773, 2787) and genes related to conjugative transfer of large self-transmissible broad-host range RP4-type IncP plasmids. The IncP transfer system comprises two regions, Tra1 and Tra2, which code for the DNA transfer and replication system (Dtr encoded by *traC* to *N*, 12 proteins) and the mating pair formation (Mpf encoded by *trbA* to *P*, 15 proteins) apparatus involved in bringing the donor and the recipient cells into intimate contact during conjugation [49]. Thus, the S1 plasmid transfer machinery is incomplete since it contains only six genes that encode TraKJI proteins involved in binding to the OriT origin of transfer, TrbJK proteins involved in the entry exclusion mechanism avoiding transfer of other plasmids of the IncP group and TrbL that is a TraG/VirB6 homolog involved in the biogenesis of the T4SS transfer pilus. Nevertheless, the pS1 plasmid is highly similar to the p3-T1 plasmid described in *Acidovorax* sp. T1, even if the p3-T1 plasmid is substantially larger (56.4 kbp) than pS1 (23 kbp). A comparison between these two plasmids revealed that all genes related to plasmid maintenance, replication and conjugation are conserved (Fig. 6), but that both plasmids diverge in genes involved in metabolic pathways. Indeed, the p3-T1 plasmid carries an operon involved in mercuric resistance and several genes that encode enzymes and genes that are related to mobile elements that do not have counterparts in pS1. At the same location as the mercury resistance genes, the pS1 sequence contains two streptomycin kinases genes, namely *straA* and *strB*, which are responsible for resistance to the streptomycin antibiotic. In bacterial isolates from plants, *strA-strB* genes are often encoded on the Tn3-type transposon Tn5393 that is generally borne on conjugative plasmids [50]. No full transposable elements were detected in the S1 putative plasmid sequence. Nevertheless, a partial Tn3-type transposon that includes the genes encoding the TniABQ transposase-related proteins is present. The functionality of the streptomycin-resistant genes present in pS1 was tested. The S1 strain was able to grow on LB plates supplemented with streptomycin concentration up to 100 μg/mL. No other tested *Dickeya* strain exhibited streptomycin resistance even at antibiotic concentrations as low as 5 μg/ml.

## Discussion

*D. fangzhongdai*, the last described *Dickeya* spp., is genomically close to the *D. dadantii*, *D. dianthicola* and *D. solani* clade [14]. Members of this clade are mainly associated to soft rot disease of herbaceous plants. However, *D. fangzhongdai* appears to have a wider range of habitats since it was reported to be not solely a soft rot pathogen of herbaceous plants, but is also connected to bleeding canker symptoms of pear trees and seems to be common in aquatic environments [9–11]. To analyse the possible links between genomic characteristics and observed diversity, we explored the signature genetic traits of the species and intra-species variability.

The comparative genomic analysis of eight *D. fangzhongdai* genomes revealed a large intra-species diversity. Indeed, the Average Nucleotide Identity values ranged from 99.9 to 96% and seven of the eight genomes can be divided into two separate clades. While the three strains of the ND14b-like cluster were all isolated from the same environment (water) in the same country, the other cluster grouped strains isolated from very different environments (water and monocot or dicot diseased plants) and from a wide geographical area (Table 1). However, a recent study on a former *Erwinia chrysanthemi* collection isolated in Japan showed that *D. fangzhongdai* strains isolated from diseased monocot plants are distributed between both clades [10, 14] indicating that genomic relatedness and habitat are not correlated.

The *D. fangzhongdai* core genome comprises over 3500 genes, a number comparable to other core genomes of the *Dickeya* species. Indeed, as determined in our Silix analysis, the size of the core genome is similar to that of the phylogenetically close *D. dianthicola* and *D. dadantii* species, for which the core genome includes 3711 (5 genomes analysed) and 3386 (4 genomes analysed) gene families, respectively. The *D. solani* core genome is larger (4009 gene families) due to the high genetic homogeneity of the *D. solani* strains sequenced so far [37, 51, 52]. This highlights the high genetic conservation of the *Dickeya* genus. Indeed, the core genome is much larger than those of other enterobacterales species, like *Escherichia coli*, that includes only around 1500 orthologous genes [53]. This high conservation is also exemplified by the fact that the vast majority of virulence genes and genes involved in the complex regulatory network that controls virulence that are well characterized in other *Dickeya* species, are also conserved in *D. fangzhongdai.*

Only a few dozen genes are specific to *D. fangzhongdai*, thereby confirming the high genetic conservation of the *Dickeya* genus. This number of species-specific gene families is rather low since our SiLix analysis revealed that other *Dickeya* species contain from 100 to 200 species-specific gene families Interestingly, all analysed *D. fangzhongdai* strains shared a few specific regulatory genes and a gene that encodes an additional pectate lyase. This pectinase belongs to the PL10 family and no members of this family were reported in the *Dickeya* genus, except in *D. chrysanthemi* Ech 1591. Pectate lyases of the family PL10 are found exclusively in a few bacteria from the plant environment, soil or human gut. *D. fangzhongdai* is the only enterobacteral species that contains a pectate lyase from PL10 family in its core genome [54]. Since some *D. fangzhongdai* strains showed higher aggressiveness and maceration potential compared to other *Dickeya* species [11], it would be interesting to analyse whether the presence of this additional pectate lyase and observed high maceration levels are connected.

*D. fangzhongdai* harbours several clusters encoding the biosynthesis of secondary metabolites that are involved in defence against stresses or the production of toxic compounds that may be important during plant-bacteria interactions. So, all analysed *D. fangzhongdai* strains harbour genes involved in the biosynthesis of zeamine, thiopeptide, cyanodactin or a NRPS/PKS cluster. Interestingly, zeamine was initially identified in the rice pathogen *D. zeae* EC1 as a phytotoxic virulence factor and is also active as an antibacterial agent [55]. In contrast to the zeamine produced by *Serratia*, the compounds produced by *D. fangzhongdai* strains have however very low nematode-killing activities [56]. Nevertheless, zeamine production does not appear to be restricted to *Dickeya* strains virulent on monocots, since *D. solani* also possesses a similar cluster [37]. Others of these genes clusters are shared by only some of the *D. fangzhongdai* strains. So, several *D. fangzhongdai* strains encode genes involved in the production of aryl polyene compounds that are structurally similar to the well-known carotenoids, and like these compounds, some of them were shown to protect the bacterium from reactive oxygen species [57]. Some strains also encode genes involved in oocydin A and a bacteriocin. Oocydin A is an anticancer haterumalide with strong antimicrobial activity against agriculturally important plant pathogenic fungi and oomycetes [58–60]. Similar cluster sequences (above 80% identity) are also present in other *Dickeya* sp., *D. solani*, various *D. dianthicola*, *D. zeae* EC1, and *D. paradisiaca* Ech703. Bacteriocins are small antibiotic-like compounds with bactericidal activity that is usually restricted to closely related species or strains, thereby increasing competition during infection [61]. They are common in Gram-negative bacteria [62]. *D. fangzhongdai* strains also encode additional NRPS/PKS complexes for which the synthesized products are not known. Other genes known to involved in bacteria-bacteria interactions and to provide a selective advantage to *D. fangzhongdai* in various environments are T5SS and T6SS effectors. It is worth to note that the C-terminal toxic parts of these effectors are very diverse between the different *D. fangzhongdai* strains (Fig. 4). All these secondary metabolites and effectors are known or predicted to harbour several biological activities such as adaptation to unfavourable environments or interspecific competition [63] and may contribute to the persistence of *D. fangzhongdai* in diverse environments.

In addition to these secondary metabolite biosynthesis pathways, intra-species *D. fangzhongdai* diversity resides mainly in the presence of genes of extrachromosomal origin since *D. fangzhongdai* strains carry diverse elements such as prophages, ICEs and plasmids. Plasmids are very rare in *Dickeya* spp. since, out of the 60 genome assemblies available in NCBI databases, only two plasmids have been identified in *Dickeya* spp.—pS1 described here and a plasmid present in *D. solani* strain 9019 that is also present in *Burkholderia* [51] and carries genes involved in antibiotic resistance. All these elements may contribute to the rapid evolution of these bacterial pathogens via horizontal gene transfer.

One of the purposes of this study was to analyse if genomic comparisons may identify traits that would differentiate strains isolated from different environments (plants versus water, monocots versus dicots) or explain the phenotypic differences reported between various *D. fangzhongdai* strains [14]. Our analyses however did not provide us with any clues to enlighten experimentally observed phenotypic differences or habitat specificity. Indeed, no genetic traits that would explain previously reported intra- and inter-species phenotype diversity [14] were identified. In the same way, we were unable to link observed differences in carbon metabolism with differences in genomic gene contents. Examination of genes that are present in JS5 and absent in all other *D. fangzhongdai* isolates (Additional file 4: Table S4) identified only 227 genes, mostly coding for hypothetical proteins (55%) and genes of extrachromosomal origin (23%). No gene clusters or metabolic pathways, that would be linked to host adaptation, could be identified. Neither were the species-specific genes abundant in regulatory or regulation-connected genes (2%).

Similarly, search for genes that are present only in *D. fangzhongdai* strains isolated from water sources did not reveal any specific genetic traits that could be associated to adaptation to the aquatic environment. Water-isolates shared only two short hypothetical genes that were not present in other *D. fangzhongdai* members.

One possible clue for explaining these differences in habitat or phenotypic capacities might be subtle variations in the regulatory networks involved in metabolic abilities, since various strain-specific genes encode regulatory proteins and furthermore, the observed high conservation of the genes involved in the virulence regulatory network does not imply a similar regulation of virulence genes. Indeed, variations in the degree of control exerted by master regulators have been observed in different *D. solani* strains with significant differences in aggressiveness [64]. Alternatively, our results may reflect the ubiquity of *D. fangzhongdai* to adapt to various environments and to infect very diverse hosts. Indeed, some *D. fangzhongdai* strains isolated from monocots were shown to be highly aggressive on dicots like potato tubers [11]. The next steps to unravel these questions would be an in-depth determination of the virulence of different *D. fangzhongdai* isolates on a large plant panel and the analysis of expression profiles in different growth conditions or during infection of diverse plant hosts.

## Conclusions

*D. fangzhongdai* isolates were found in different habitats like monocot and dicot plants and waterways. Comparison of the genome information of eight members of this new species isolated from these diverse environments revealed a high proportion of species core genome genes (three quarters of total genomes). These include the majority of virulence genes and virulence-related global regulators characterized in other *Dickeya* species. Importantly, it also allows the identification of a few dozen genes specific to *D. fangzhongdai* that provide a basis for the development of DNA-based effective detection and diagnosis.

The intra-species diversity of the *D. fangzhongdai* species resides mainly in secondary metabolites biosynthetic pathways, T5SS and T6SS-related toxins or the repertoire of genes of extrachromosomal origin and also in gene clusters related to metabolism and transport. However, no genetic traits that would differentiate strains isolated from different environments and contribute to habitat specificity could be identified. This might reflect the ubiquity of *D. fangzhongdai* to adapt to various environments including fresh water and infect very diverse hosts. *D. fangzhongdai* may thus spread via waterways and constitute a potential threat to several economically important crops.

## Additional files

Additional file 1: **Figure S1.** *Dickeya* strains used in comparative genomics. (DOCX 1173 kb)

Additional file 2: **Table S1.** T5SS and T6SS effectors present in the different *Dickeya fangzhongdai* genomes. (DOCX 25 kb)

Additional file 3: **Tables S2** and **S3.** Genes found in each of the JS5 and Nd14b genomic clades that are absent from the other *Dickeya fangzhongdai* genomes. (ZIP 37 kb)

Additional file 4: **Table S4.** Genes present in the JS5 strain that are absent in all other *Dickeya fangzhongdai* strains. (XLSX 17 kb)

## Abbreviations

ANI: Average Nucleotide Identity
bp: Base pairs
CDI: Contact-dependent immunity
DDH: In silico DNA-DNA Hybridization
ICE: Integrative and conjugative element
NRPS: Non-ribosomal peptide synthase
PKS: Polyketide synthase
rhs: Recombination hot spot
TxSS: Type x secretion system
