# Supplementary material for: Genomic characterisation of the new Dickeya fangzhongdai species regrouping plant pathogens and environmental isolates
Source: BMC Genomics. 2019 Jan 11;20:34. doi: 10.1186/s12864-018-5332-3 (PMC6329079; doi:10.1186/s12864-018-5332-3)
Supplement: Supplementary file 1 — Figure S1. Dickeya strains used in comparative genomics. (DOCX 1173 kb) [file 12864_2018_5332_MOESM1_ESM.docx]

**Genomic characterisation of the new *Dickeya fangzhongdai* species regrouping plant pathogens and environmental isolates**

Špela Alič^a,b^, Jacques Pedron^c^, Tanja Dreo^a^, Frédérique Van Gijsegem^c, *^

^a^National Institute of Biology, Vecna pot 111, SI-1000 Ljubljana, Slovenia;

^b^Jozef Stefan International Postgraduate School, Jamova 39, SI-1000 Ljubljana, Slovenia;

^c^Institut d’Ecologie et des Sciences de l’Environnement de Paris, Sorbonne Universités, UPMC Univ Paris 06, Diderot Univ Paris 07, UPEC Univ Paris 12, CNRS, INRA, IRD, 4 Place Jussieu, 75005, Paris, France

Email addresses: Spela.Alic@nib.si, [jacques.pedron@upmc.fr](mailto:jacques.pedron@upmc.fr), tanja.dreo@nib.si, [vangijse@agroparistech.fr](mailto:vangijse@agroparistech.fr)

*Corresponding author: [vangijse@agroparistech.fr](mailto:vangijse@agroparistech.fr)


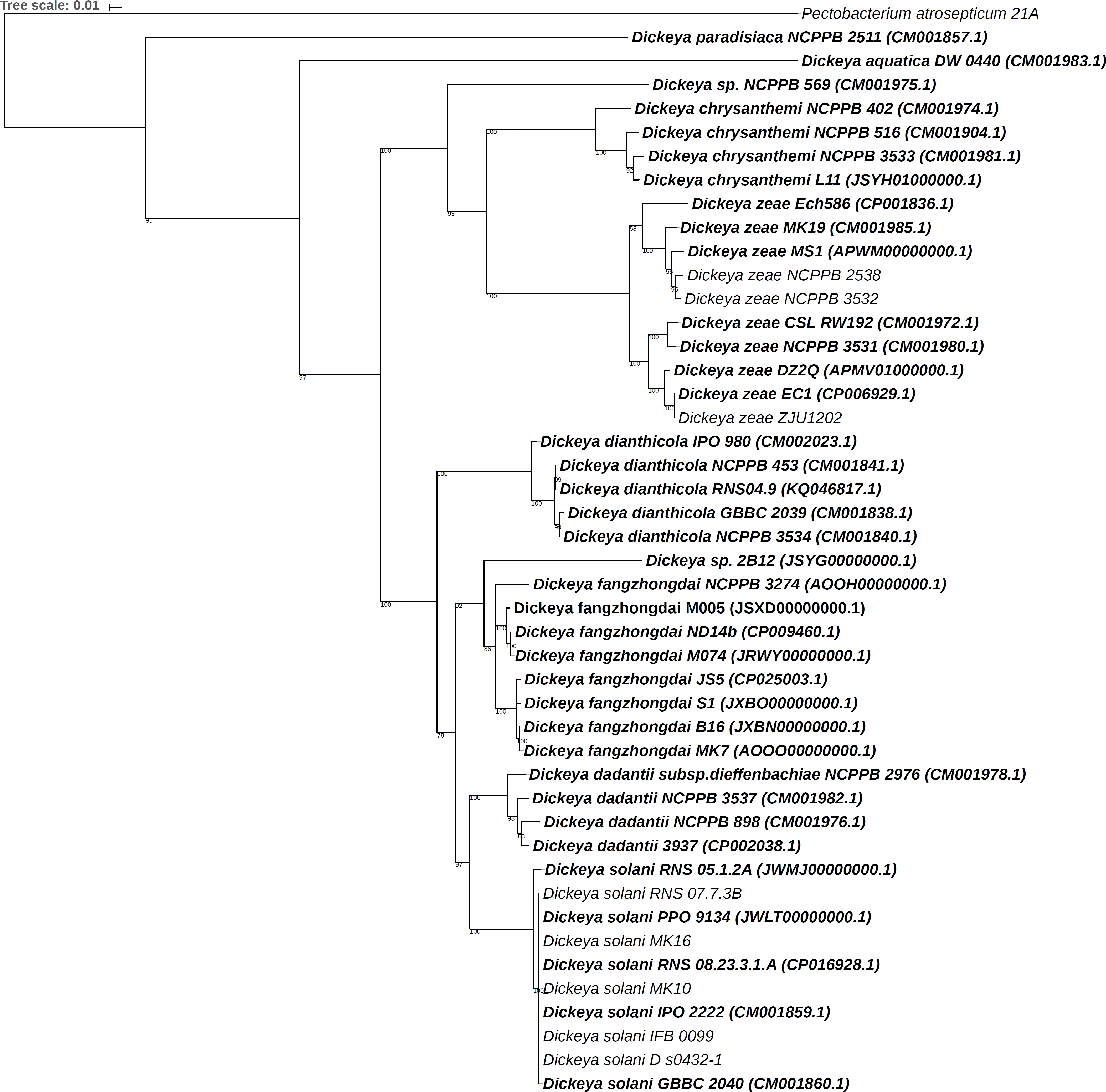


Figure S1: Consensus tree of the concatenated sequences (total 8274 bp) of purA, dnaN, gapA, gyrA, atpD and rpoS of 44 members of the genus *Dickeya*. Strains written in bold with corresponding GenBank accession number were included in the SiLix based comparative genomics analysis.

The starting tree was created with the BIONJ algorithm. Bootstrap percentages were calculated with 1000 replicates. *Pectobacterium atrosepticum* 21A was used as an out-group to root the phylogenetic tree.
