# Supplementary material for: Genomic characterisation of the new Dickeya fangzhongdai species regrouping plant pathogens and environmental isolates
Source: BMC Genomics. 2019 Jan 11;20:34. doi: 10.1186/s12864-018-5332-3 (PMC6329079; doi:10.1186/s12864-018-5332-3)
Supplement: Supplementary file 2 — Table S1. T5SS and T6SS effectors present in the different Dickeya fangzhongdai genomes. (DOCX 25 kb) [file 12864_2018_5332_MOESM2_ESM.docx]

**Genomic characterisation of the new *Dickeya fangzhongdai* species regrouping plant pathogens and environmental isolates**

Špela Alič^a,b^, Jacques Pedron^c^, Tanja Dreo^a^, Frédérique Van Gijsegem^c, *^

^a^National Institute of Biology, Vecna pot 111, SI-1000 Ljubljana, Slovenia;

^b^Jozef Stefan International Postgraduate School, Jamova 39, SI-1000 Ljubljana, Slovenia;

^c^Institut d’Ecologie et des Sciences de l’Environnement de Paris, Sorbonne Universités, UPMC Univ Paris 06, Diderot Univ Paris 07, UPEC Univ Paris 12, CNRS, INRA, IRD, 4 Place Jussieu, 75005, Paris, France

Email addresses: Spela.Alic@nib.si, [jacques.pedron@upmc.fr](mailto:jacques.pedron@upmc.fr), tanja.dreo@nib.si, [vangijse@agroparistech.fr](mailto:vangijse@agroparistech.fr)

*Corresponding author: [vangijse@agroparistech.fr](mailto:vangijse@agroparistech.fr)

Table S1: T5SS and T6SS effectors present in the different *Dickeya fangzhongdai* genomes and compared to *Dickeya dadantii 3937* genome

1. T5SS :

- Tsp / Hec

|  | **Genes and its RAST IDs** | | |
| --- | --- | --- | --- |
| **Dickeya strain** | ***hrpN*** | ***tspB (hecB)*** | ***tspA (hecA2)*** |
| *D. dadantii* 3937 | 2554 | 2551 | 2548 |
| *D. fangzhongdai* S1 | 2931 | 2934 | contig limit, 2935 truncated across the limit |
| *D. fangzhongdai* MK7 | 2501 | 2499 | absent, synteny ok |
| *D. fangzhongdai* B16 | 2936 | 2938 | contig limit |
| *D. fangzhongdai* M005 | 3393 | 3396 | contig limit, 3550 truncated across the limit |
| *D. fangzhongdai* JS5 | 2533 | 2930 | 2529 |
| *D. fangzhongdai* M074 | 4145 | 4148 | contig limit, 4265 truncated across the limit |
| *D. fangzhongdai* NCPPB 3274 | 2610 | 2608 | 2605 truncated |
| *D. fangzhongdai* ND14b | 126 | 128 | 130 |

- Cdi

|  | **Gene and its RAST IDs** |
| --- | --- |
| **Dickeya strain** | ***tspA (hecA1)*** |
| *D. dadantii* 3937 | 2458 |
| *D. fangzhongdai* S1 | contig limit, 3342 truncated |
| *D. fangzhongdai* MK7 | 2408 and 2409, splitted |
| *D. fangzhongdai* B16 | contig limit, 1853 truncated |
| *D. fangzhongdai* M005 | contig limit, 4549 truncated |
| *D. fangzhongdai* JS5 | 2432 and 2437, splitted |
| *D. fangzhongdai* M074 | contig limit, 4540 truncated across the milit |
| *D. fangzhongdai* NCPPB 3274 | 2512 and 2513, splitted, truncated |
| *D. fangzhongdai* ND14b | 216 |

1. T6SS

- RhsA

|  | **Genes and its RAST IDs** | | |
| --- | --- | --- | --- |
| **Dickeya strain** | ***hcpA*** | ***vgrGa*** | ***rhsA*** |
| *D. dadantii* 3937 | 905 | 904 | 902 |
| *D. fangzhongdai* S1 | - | - | - |
| *D. fangzhongdai* MK7 | - | - | - |
| *D. fangzhongdai* B16 | - | - | - |
| *D. fangzhongdai* M005 | - | - | 2493 contig limit |
| *D. fangzhongdai* JS5 | - | - | - |
| *D. fangzhongdai* M074 | - | - | - |
| *D. fangzhongdai* NCPPB 3274 | - | - | - |
| *D. fangzhongdai* ND14b | - | - | - |

- RhsC

|  | **Genes and its RAST IDs** | | | | |
| --- | --- | --- | --- | --- | --- |
| **Dickeya strain** | ***hcpC*** | ***vgrGc*** | ***rhsC*** | ***rhsI_C1_*** | ***rhsI_C2_*** |
| *D. dadantii* 3937 | 1522 | 1523 | 1529 | 1531 | 1532 |
| *D. fangzhongdai* S1 | 2060 | 2059 | 2052 | 2048 | absent |
| *D. fangzhongdai* MK7 | 1424 | 1426 | 1433 | 1434 | absent |
| *D. fangzhongdai* B16 | 2427 | 2425 | contig limit | ? | ? |
| *D. fangzhongdai* M005 | 379 | 378 | 371 | 370 | 369 |
| *D. fangzhongdai* JS5 | 1526 | 1528 | 1535 | 1539 | 1540 |
| *D. fangzhongdai* M074 | 3814 | contig limit 3813 | 1050 truncated | 1052 | absent |
| *D. fangzhongdai* NCPPB 3274 | 1575 | 1576 | splitted 1581, 1582 | 1583 | absent |
| *D. fangzhongdai* ND14b | 1125 | 1124 | 1118 | 1115 | 1113 |

- RhsB

|  | **Genes and its RAST IDs** | | | |
| --- | --- | --- | --- | --- |
| **Dickeya strain** | ***hcpB*** | ***vgrGb*** | ***rhsB*** | ***rhsI_C1_*** |
| *D. dadantii* 3937 | 3049 | 3048 | 3046 | 3045 |
| *D. fangzhongdai* S1 | contig limit 2443 | contig limit | truncated 3481 | absent |
| *D. fangzhongdai* MK7 | - | - | - | - |
| *D. fangzhongdai* B16 | - | - | - | - |
| *D. fangzhongdai* M005 | - | - | - | - |
| *D. fangzhongdai* JS5 | - | - | - | - |
| *D. fangzhongdai* M074 | - | - | - | - |
| *D. fangzhongdai* NCPPB 3274 | 3123 | 3122 | 3120 | 3119 |
| *D. fangzhongdai* ND14b | - | - | - | - |

- rhsF

|  | **Genes and its RAST IDs** | | |
| --- | --- | --- | --- |
| **Dickeya strain** | **hcpF** | **vgrGf** | **rhsF** |
| *D. dadantii* 3937 |  |  |  |
| *D. fangzhongdai* S1 | contig limit 676 | contig limit 3478 | 4009 truncated |
| *D. fangzhongdai* MK7 | 4529 | 4528 | 4526 |
| *D. fangzhongdai* B16 | 3808 | 3807 | 3805 |
| *D. fangzhongdai* M005 | contig limit 837 | - | contig limit 836 |
| *D. fangzhongdai* JS5 | 57 | 58 | 60 |
| *D. fangzhongdai* M074 | 2999 | 3000 | 3002 |
| *D. fangzhongdai* NCPPB 3274 | 4711 | 4709 | 4707 |
| *D. fangzhongdai* ND14b | 2498 | 2497 | 2495 |
